# Endobronchial Metastasis From Osteosarcoma in an Adolescent: A Rare Case Report

**DOI:** 10.1111/1759-7714.70355

**Published:** 2026-07-18

**Authors:** Yanling He, Yanping Chen, Yanhong Liu, Juyan Chen, Ahmad Muhammad, Huiwen Jin, Yi Xiao

**Affiliations:** ^1^ Yan'an Hospital Affiliated to Kunming Medical University Kunming Yunnan China; ^2^ Kunming Medical University Kunming Yunnan China

**Keywords:** bronchial, endobronchial, metastasis, osteosarcoma, pulmonary

## Abstract

A 14‐year‐old female was diagnosed with osteosarcoma of the left proximal tibia and underwent neoadjuvant chemotherapy followed by wide local resection and limb‐sparing surgery. Thirteen months after surgery, she developed coughing, wheezing, and shortness of breath. Chest CT revealed stenosis in the right main and middle bronchi with a soft‐tissue density lesion. No suspicious metastatic lesions were observed around the airways, and the analysis of bronchoscopic biopsy specimens confirmed a rare endobronchial metastasis of osteosarcoma. Despite bronchoscopic intervention, the patient eventually died due to late detection and rapid tumor progression. These findings suggest that early bronchoscopic assessment of the airway mucosa is critical.

## Graphical abstract

Endobronchial metastasis from osteosarcoma is rare. This case of a 14‐year‐old female with tibial osteosarcoma demonstrates an atypical metastatic pathway to the bronchus. She developed respiratory symptoms 13 months after initial treatment, and subsequent bronchoscopic biopsies confirmed endobronchial metastasis. However, due to rapid tumor progression and late diagnosis, the patient eventually died. This case highlights that early invasive changes of the airway mucosa, which are often detectable by bronchoscopy but not apparent on chest CT.

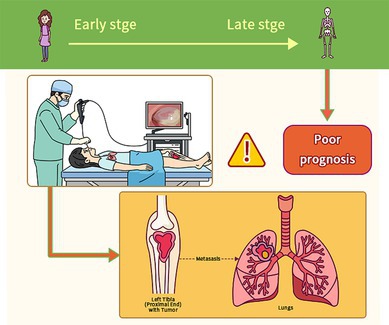

## 1 Introduction

Osteosarcoma (OS) is an aggressive tumor, often leading to distant metastasis, which occurs in 15%–40% of patients [1, 2]. Almost all deaths in OS patients are attributed to distant metastasis [3, 4]. Once metastasis develops, survival outcomes significantly worsen, with the 5‐year survival rate dropping to 20% [5, 6]. The lungs are the most common site for metastasis, and this pulmonary tropism may be explained by the lung's capillary bed, where alveoli trap OS cells, and by the primary OS tumor conditioning the lung as a pre‐metastatic niche [7]. However, metastasis of OS to endobronchial is rare. This report describes a rare case of endobronchial metastasis in an adolescent female to highlight diagnostic and clinical considerations.

## 2 Case

A 14‐year‐old female was diagnosed with osteosarcoma (OS) of the left proximal tibia. Four months prior to admission, she underwent wide local resection with limb‐sparing surgery and knee joint replacement. Postoperative pathological examination confirmed periosteal OS with negative surgical margins.

She received one cycle of the MAP regimen (high‐dose methotrexate, cisplatin, and doxorubicin) before surgery and two cycles afterward. However, the patient developed severe oral mucositis, nausea, and vomiting, which she tolerated poorly during treatment; therefore, the protocol was switched to ifosfamide alone for the subsequent four cycles. One month after surgery, follow‐up CT scans of the lower extremity and chest showed no metastatic lesions. Subsequent CT imaging was conducted every 3 months. Six months postoperatively, cranial CT and bone scans were added to routine chest and lower extremity CT, with the most recent evaluation performed approximately 9 months after surgery. No obvious recurrence was observed on serial CT imaging during the subsequent year, and the patient resumed normal life but did not adhere to recommended 12th‐month postoperative follow‐up.

Thirteen months after surgery, she was admitted to the hospital with a 1‐week history of cough, wheezing, and shortness of breath. Chest CT revealed stenosis of the right main and middle bronchi, with an associated soft‐tissue‐density mass (Figure 1A). A suspicious metastatic lesion was also noted adjacent to the left pleura (Figure 1B). Bronchoscopy (Figure 2A,B) revealed a neoplasm obstructing the right main bronchus, causing severe stenosis. The mass was successfully resected using laser therapy, achieving effective hemostasis (Figure 2C). Histopathological analysis confirmed a malignant lesion consistent with metastatic OS. Although further genetic testing and biopsy of the left upper lobe lesion were recommended, her parents declined, and she was discharged 8 days later. She subsequently received three cycles of the IE regimen (irinotecan and etoposide) in the oncology department of another hospital.

**FIGURE 1:**
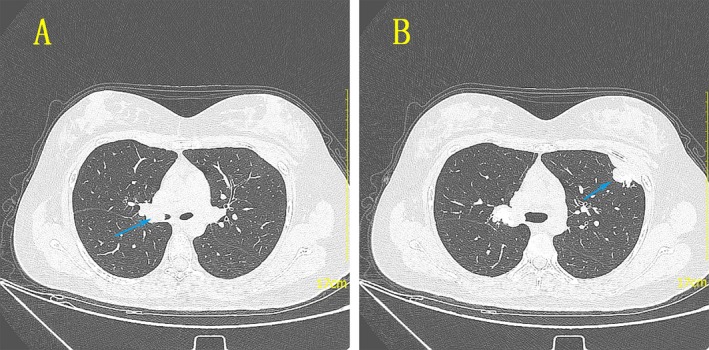
Chest CT (axial view, lung window) demonstrating (A) soft‐tissue density lesion causing stenosis of the right main bronchus (blue arrow) and (B) a secondary metastatic nodule adjacent to the left pleura (blue arrow).

**FIGURE 2:**
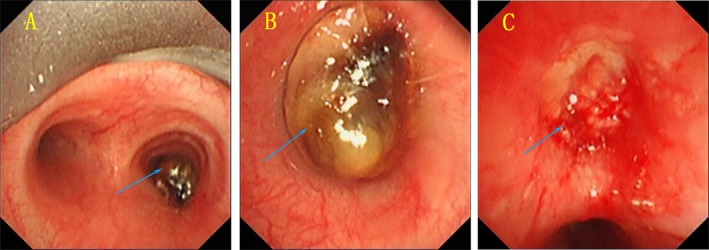
The bronchoscope showing neoplasm located in the right main bronchus (A, B), and the bronchial mass resected after bronchoscopy (blue arrow) (C).

Four months later, she was readmitted due to worsening dyspnea. Physical examination revealed a respiratory rate of 50 breaths/min and a heart rate of 155 beats/min. Chest CT indicated that the right main bronchus was completely obstructed (Figure 3A), and the soft tissue nodule in the upper lobe of the left lung had significantly increased in size (Figure 3B). Bronchoscopic treatment was attempted again; however, the mass had hardened considerably, and complete resection would have required a prolonged procedure that the patient was unlikely to tolerate. Thus, the right main bronchus remained completely obstructed (Figure 4A,B).

**FIGURE 3:**
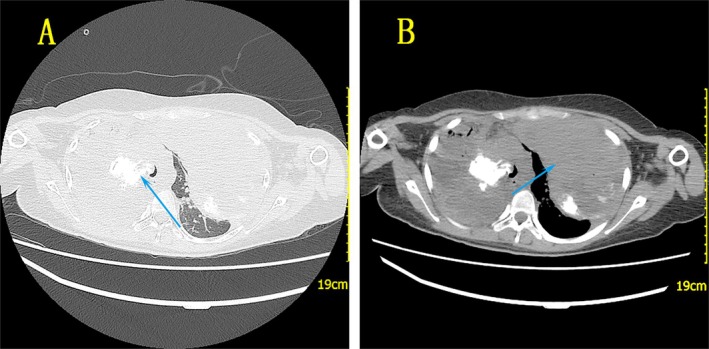
Follow‐up chest CT (axial view, lung window) displaying the neoplasm and stenosis in the main bronchus and right main bronchus is completely blocked (blue arrow) (A). The soft tissue nodule is much larger in the upper lobe of the left lung (blue arrow) (B).

**FIGURE 4:**
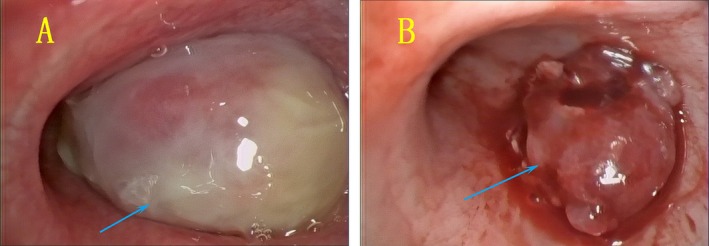
Bronchoscopic images showing (A) near‐total tracheal obstruction (~95%) and (B) persistent right main bronchus obstruction after partial resection, with the carina and left main bronchus visible (blue arrow).

A multidisciplinary consultation was held. Given the patient's unstable vital signs indicating high anesthetic‐related mortality risk, and parental refusal for surgery, no further invasive procedures were conducted. Ultimately, the patient died of respiratory failure 23 days later.

## 3 Discussion and Conclusion

Endobronchial metastases account for approximately 1.1% of all endobronchial malignancies [8]. To our best knowledge, comparable cases in the literature are extremely limited. Existing similar cases primarily presented with extrapulmonary recurrence or airway tumors occurring near suspected pulmonary metastatic lesions [9, 10]. In this case, the tumor occurred in the right airway, whereas chest CT revealed no obvious metastatic lesions near the right airway but identified a suspicious metastatic lesion on the left pleura. We therefore speculate that this case represents a rare pattern of endobronchial metastasis. We hypothesize that the endobronchial tumor originated from lymphatic spread, with malignant cells forming solid tumor masses beneath the bronchial epithelium, eventually protruding into the lumen.

Additionally, the cause of rapid growth and progressive hardening of the airway tumor remains unclear. Chemotherapy may induce atypical metastatic patterns and alter disease progression [11], potentially selecting more aggressive variants with greater metastatic potential [12, 13]. Furthermore, OS may continue to progress despite treatment [14].

Early diagnosis of endobronchial metastasis may improve treatment outcomes. Regular follow‐up is of paramount importance. However, invasive changes in the airway mucosa are difficult to detect on CT scans at an early stage, resulting in missed diagnoses. Thereby, bronchoscopy is crucial for comprehensive airway evaluation and guiding surgical intervention.

For inoperable patients, novel therapeutic therapies may offer potential benefits. Recent studies indicate that microRNA‐605‐3p and ezetimibe may have therapeutic effects on OS [15]. Further research and animal studies might validate their clinical applications. Additionally, targeted therapies involving different molecular pathways have emerged as promising research areas, indicating a broader shift toward precision medicine [16, 17].

This case is of great value, but also has limitations. A PET scan was not performed due to financial constraints. However, when feasible, PET scans should be routinely employed for surveillance during follow‐up. Earlier bronchoscopy and more extensive follow‐up might have altered the clinical outcome.

In conclusion, although rare, bronchial metastasis should be considered in the differential diagnosis of dyspnea. Bronchoscopy plays a critical role in the diagnosis, early management of airway tumors, staging assistance, and assessment of surgical options. Routine pulmonary CT imaging combined with PET‐CT is also essential for ongoing surveillance.

## Author Contributions

**Yanhong Liu:** resources, project administration. **Juyan Chen:** resources, visualization. **Yanling He:** conceptualization, writing – original draft, writing – review and editing. **Ahmad Muhammad:** writing – review and editing, data curation. **Yanping Chen:** resources, writing – review and editing, investigation. **Yi Xiao:** supervision, conceptualization, writing – review and editing. **Huiwen Jin:** writing – review and editing, investigation.

## Funding

The authors have nothing to report.

## Ethics Statement

This study was conducted following the Helsinki Declaration II and was approved by the Institutional Review Boards of Yan'an Hospital affiliated to Kunming Medical University.

## Consent

Consent was obtained from the legal guardian for the publication of this case report and accompanying images.

## Conflicts of Interest

The authors declare no conflicts of interest.

## Data Availability

The datasets generated and/or analyzed during the current study are not publicly available due to patient privacy but are available from the corresponding author on reasonable request.
